# Impact of human papillomavirus-related genital diseases on quality of life and psychosocial wellbeing: results of an observational, health-related quality of life study in the UK

**DOI:** 10.1186/1471-2458-13-1065

**Published:** 2013-11-12

**Authors:** Géraldine Dominiak-Felden, Catherine Cohet, Samantha Atrux-Tallau, Hélène Gilet, Amanda Tristram, Alison Fiander

**Affiliations:** 1 Department of Epidemiology, Sanofi Pasteur MSD, Lyon, France; 2 Mapi, Lyon, France; 3 Obstetrics and Gynaecology, Wales College of Medicine, Cardiff University, Cardiff, UK

**Keywords:** Cervical neoplasia, Genital warts, HPV, Vaccination, Vulval disease

## Abstract

**Background:**

Data on the psychosocial burden of human papillomavirus (HPV)-related diseases other than cervical cancer are scarce. The objectives of this study were to measure and compare the psychosocial burden and the impact on health-related quality of life (HRQoL) of HPV-related lower genital tract diseases and genital warts (GW) using several generic and disease-specific instruments.

**Methods:**

Overall, 842 individuals with normal cervical cytology (n = 241), borderline nuclear abnormalities and/or mild dyskaryosis (n = 23), cervical intraepithelial neoplasia (CIN)1 (n = 84), CIN2/3 (n = 203), vulval intraepithelial neoplasia (VIN)2/3 (n = 43), GW (n = 186) and a history of GW (non-current) (n = 62) were included. The generic European Quality of Life Index Version 5D (EQ-5D) questionnaire was completed by patients with GW and VIN2/3. Sexual functioning was evaluated using the Change in Sexual Functioning Questionnaire (CSFQ). Psychosocial impact was measured in women using the HPV Impact Profile (HIP) questionnaire. HRQoL was assessed using a GW-specific questionnaire, the Cuestionario Especifico en Condilomas Acuminados (CECA) (completed by patients with GW and history of GW). For each instrument, scores were compared between groups using the Student's *t*-test. In addition, utility loss due to GW and VIN2/3 was evaluated by comparing mean EQ-5D scores weighted by age and sex with the UK general population normal values.

**Results:**

A significant psychosocial impact was found in women diagnosed with HPV-related genital diseases, particularly in those with GW. The health state of younger adults with GW was significantly impaired compared with UK normal values (mean EQ-5D index score 0.86 vs 0.94, p < 0.001 for 18–24-year-olds; 0.87 vs 0.93, p = 0.030 for 25–34-year-olds). VIN2/3 was found to have a significant negative impact on sexual functioning, and women with VIN2/3 had a highly impaired health state compared with women in the UK general population (weighted mean EQ-5D index score 0.72 vs 0.89, p < 0.001; weighted mean Visual Analogue Scale score 62 vs 85, p < 0.001).

**Conclusions:**

HPV-related lower genital tract lesions and GW significantly impair psychosocial wellbeing and HRQoL. The psychosocial aspects of HPV-related diseases need to be considered when evaluating the potential benefit of HPV vaccination.

## Background

Approximately 70% of sexually active individuals will be infected with human papillomavirus (HPV) during their lifetime [1]. Most infections are asymptomatic and clear spontaneously [2], but persistent infections with some HPV types may lead to anogenital neoplasia and genital warts (GW) [3,4].

The main burden of HPV-related diseases is due to cervical cancer. HPV-related precancerous lesions of the lower genital tract (e.g. cervical intraepithelial neoplasia [CIN] grades 1/2/3 and vulval intraepithelial neoplasia [VIN] grades 1/2/3) are also potentially serious conditions, requiring repeated health care visits for disease monitoring and treatment. Over the last 40 years, the incidence of VIN and vulval cancer has increased in developed countries, predominantly among women aged <50 years [5-8]. The incidence of GW has also increased considerably in many European countries, including the UK [9,10]. GW are unsightly and often cause discomfort, and only a minority of cases resolve without treatment. Treatment is usually lengthy and painful, and often does not prevent recurrences [3]. The increases in incidence of VIN, vulval cancer and GW may be partially explained by increased HPV transmission and infection rates due to changes in behaviour [11,12].

In Europe, two HPV genotypes (16 and 18) are responsible for approximately 73% of cervical cancers [13] and the majority of HPV-related vulval and vaginal cancers [14]. HPV 6 and 11 are responsible for 90% of GW affecting both men and women [3]. Primary prevention of HPV-related diseases through HPV vaccination is recommended in many countries. Two prophylactic HPV vaccines, Gardasil® (Sanofi Pasteur MSD, Lyon, France) and Cervarix® (GlaxoSmithKline Biologicals, Rixensart, Belgium), are available, which both protect against precancerous lesions, including CIN1/2/3, VIN2/3, and cervical cancer caused by HPV 16 and 18. In addition, the quadrivalent vaccine Gardasil® also protects against infection and disease caused by HPV 6 and 11, including GW. Determining the impact of HPV-related disease on patients’ health-related quality of life (HRQoL) is important to fully assess the value of HPV vaccination. Many studies have documented the psychosocial burden associated with cervical cancer and its impact on HRQoL [15-19] and some studies have evaluated the impact of GW [20-25]. However, fewer studies have quantified the impact of other HPV-related diseases [26-30] or used an HPV-specific questionnaire.

The objective of the Papillomavirus ASsociated QUAlity of Life (PasQual) study was to assess the psychosocial burden and impact on HRQoL of HPV-related lower genital tract diseases and GW in the UK using generic and disease-specific instruments.

## Methods

### Participants

Study participants were aged 18–64 years and included women with normal cervical cytology, borderline nuclear abnormalities and/or mild dyskaryosis, CIN1, CIN2/3 and VIN2/3, and women and men with GW or with a history of GW. Participants included in the GW group were recruited any time during a current episode of GW (i.e. undergoing diagnosis and/or treatment of a GW episode that was clinically present at the time of inclusion in the study), whether this was a first episode or a recurrence. Participants included in the history of GW group were attending health care facilities for the follow-up of a previous sexually transmitted infection (STI) and had previously experienced an episode of GW that had resolved ≥6 months before study enrolment. Participants were made aware of their diagnosis and provided written informed consent prior to administration of questionnaires. Exclusion criteria included any concomitant STI, any concomitant condition that might have an impact on the psychosocial burden of participants, and vaccination with an HPV vaccine.

### Study design and procedures

This was a multicentre, observational, cross-sectional study. Participants were recruited at 15 centres (six secondary care colposcopy and gynaecology clinics, five genitourinary medicine clinics, two general practice clinics and two family planning clinics) across the UK between May 2008 and March 2009.

The protocol and other study documents were approved by the Multi-Centre Research Ethics Committee for Wales (reference 07/MRE09/75), and local institutional approvals were obtained as appropriate. The study was conducted in accordance with the Declaration of Helsinki, Good Epidemiological Practice guidelines [31], and local regulations.

Basic demographic data, including age, sex, socioeconomic status, current marital/relationship status, medical history and clinical status, were collected at enrolment. Four instruments were used to measure the patient’s perspective: two disease-specific questionnaires (the HPV Impact Profile [HIP] questionnaire [32] and the Cuestionario Especifico en Condilomas Acuminados [CECA] [33,34] and two generic questionnaires (the European Quality of Life Index Version 5D [EQ-5D] [35] and the Change in Sexual Functioning Questionnaire [CSFQ] [36]). The instruments used in each HPV disease group are shown in Table 1.

**Table 1:** Patient-reported outcome instruments used in each group

**Group**	**Patient-reported outcome instrument**			
	**HIP**	**CSFQ**	**EQ-5D**	**CECA**
Normal cervical cytology	✓	✓		
Borderline nuclear abnormalities and/or mild dyskaryosis	✓	✓		
CIN1/2/3	✓	✓		
VIN2/3	✓	✓	✓	
GW (women)	✓	✓	✓	✓
History of GW (women)	✓	✓		✓
GW (men)		✓	✓	✓
History of GW (men)		✓		✓

CECA, Cuestionario Especifico en Condilomas Acuminados; CIN, cervical intraepithelial neoplasia; CSFQ, Change in Sexual Functioning Questionnaire; EQ-5D, European Quality of Life Index Version 5D; GW, genital warts; HIP, Human Papillomavirus Impact Profile; VIN, vulval intraepithelial neoplasia.

Ticks indicate that a given patient-reported outcome instrument was administered.

The sample size calculation was based on the results of the US validation study for the HIP questionnaire [32], considering an anticipated response rate of 60% [37], a two-sided *t*-test with 90% power to detect a statistically significant difference in HIP scores between the CIN group and the GW group at baseline, and study feasibility considerations. A sample of 50 participants with VIN2/3 and 200 in each of the other groups was defined.

### Patient-reported outcome instruments

#### HIP

The HIP questionnaire is specifically designed to measure psychosocial burden in women with HPV-related diseases and comprises 29 items grouped into seven psychosocial dimensions: worries and concerns; emotional impact; sexual impact; self-image; partner issues and transmission; interactions with doctors; and health control and life impact [32]. Dimension scores and a total burden score, all ranging from 0 to 100, are calculated based on item scores. Higher scores indicate a greater psychosocial impact of HPV.

#### CSFQ

Short versions of the CSFQ, a validated, sex-specific questionnaire designed to measure the impact of diseases and medication on sexual functioning, were used [36], comprising 14 items grouped into five dimensions: pleasure, desire/frequency, desire/interest, arousal/excitement, and orgasm. Dimension scores and a total sexual functioning score are calculated as the sum of item scores. Lower scores indicate worse sexual functioning.

#### EQ-5D

The EQ-5D is a self-administered, generic, preference-based instrument designed to measure the impact of disease on the general health state, and comprises the EQ-5D index and the EQ Visual Analogue Scale (VAS) [35]. The EQ-5D index requires participants to state the extent of problems in five dimensions—mobility, self-care, usual activities, pain/discomfort and anxiety/depression—with a higher EQ-5D index score indicating a better general health state. The EQ VAS requires participants to rate their current state of health on a scale of 0–100, with 0 indicating the worst imaginable and 100 the best imaginable health state.

#### CECA

The CECA is a validated, ten-item, self-administered questionnaire designed specifically to measure the HRQoL of individuals with GW [33,34]. CECA items are grouped into two dimensions: emotional (six items) and sexual (four items); a global score is derived from the ten item scores. Scores range from 0 (worst HRQoL) to 100 (best HRQoL).

### Data analysis

Women with normal cervical cytology were considered as the reference group against which women with HPV-related diseases were compared. As the CECA is specific to GW, healthy participants having never experienced GW are not able to answer the questionnaire and are not a suitable comparator. Therefore, in the assessment of CECA scores, participants with a history of GW were used as the reference group. The Student’s *t*-test (HIP, CSFQ and CECA) and analysis of variance (HIP and CSFQ) were used for between-group comparisons of questionnaire scores. Crude EQ-5D scores were described for participants with GW and participants with VIN2/3. Weighted mean EQ-5D scores were compared with UK general population normal values [38] using the Student’s *t*-test (data weighted according to the age/sex distribution of the UK population). Age-stratified comparisons of EQ-5D scores between participants with GW and UK general population norms were also conducted; due to low numbers of participants with VIN2/3, age stratified data for VIN2/3 are not presented. A p value ≤0.05 was considered statistically significant. All statistical analyses were performed using SAS software (version 9.1.3, SAS Institute, Inc., Cary, NC, USA).

## Results

### Study population

**Figure 1:**
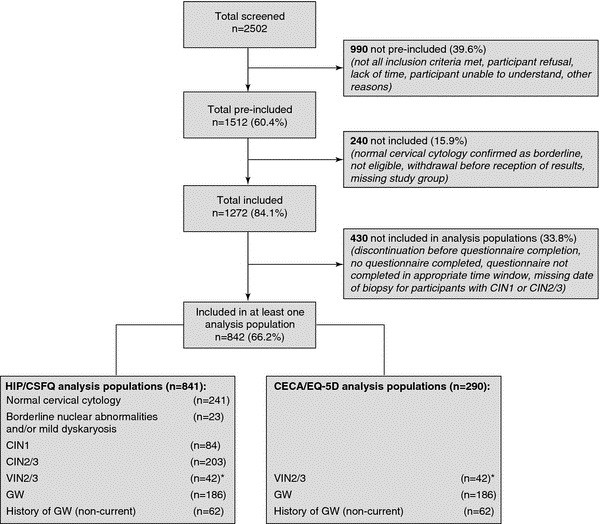
**Flow chart of study populations.** CECA, Cuestionario Especifico en Condilomas Acuminados; CIN, cervical intraepithelial neoplasia; CSFQ, Change in Sexual Functioning Questionnaire; EQ-5D, European Quality of Life Index Version 5D; GW, genital warts; HIP, Human Papillomavirus Impact Profile; VIN, vulval intraepithelial neoplasia. * Of the 43 patients in the VIN2/3 group, one patient did not complete at least one item of the HIP/CSFQ questionnaires; a different patient did not complete at least one item of the CECA/EQ-5D questionnaires.

Of the 2502 individuals screened for the PasQual study, 1512 (60.4%) met all the screening criteria and 1272 (50.8%) were included in the study after confirmation of diagnosis. Of these, 842 (66.2%) completed at least one item of one or more questionnaires. Patients with VIN2/3, GW and history of GW completed at least one item of the EQ-5D or the CECA (n = 290). Overall, numbers in each group were as follows: normal cervical cytology, n = 241; borderline nuclear abnormalities and/or mild dyskaryosis, n = 23; CIN1, n = 84; CIN2/3, n = 203; VIN2/3, n = 43; GW, n = 186; and history of GW, n = 62 (Figure 1). The mean age of study participants was 34 years (range 18–64 years); the socio-demographic characteristics of study participants are shown in Table 2.

**Table 2:** Socio-demographic characteristics of study participants

	**Normal (n = 241)**	Borderline* (n = 23)	**CIN1 (n = 84)**	**CIN2/3 (n = 203)**	**VIN2/3 (n = 42)**	**GW (n = 186)**	**GW history (n = 62)**	**Total (n = 841)**
Age in years								
Mean (SD)	40.4 (10.1)	35.6 (9.0)	32.9 (9.6)	31.7 (8.1)	44.8 (10.0)	28.0 (9.2)	28.4 (7.8)	34.0 (10.6)
Sex, n (%)								
Female	241 (100.0)	23 (100.0)	84 (100.00)	203 (100.0)	42 (100.0)	101 (54.3)	39 (62.9)	733 (87.2)
Male	0	0	0	0	0	85 (45.7)	23 (37.1)	108 (12.8)
Level of education, n (%)								
Secondary education (5–7years of education)	109 (45.2)	8 (34.8)	25 (29.8)	66 (32.5)	17 (40.5)	57 (30.6)	17 (27.4)	299 (35.6)
Further education (≥8 years of education)	132 (54.8)	15 (65.2)	58 (69.0)	137 (67.5)	23 (54.8)	129 (69.4)	45 (72.6)	539 (64.1)
Other†	0	0	1 (1.2)	0	2 (4.8)	0	0	3 (0.4)
Employment status, n (%)								
Employed full-time	95 (39.4)	12 (52.2)	42 (50.0)	104 (51.2)	16 (38.1)	97 (52.2)	38 (61.3)	404 (48.0)
Employed part-time	63 (26.1)	5 (21.7)	15 (17.9)	30 (14.8)	8 (19.0)	14 (7.5)	3 (4.8)	138 (16.4)
Retired	11 (4.6)	0	0	0	2 (4.8)	0	0	13 (1.5)
Student	4 (1.7)	1 (4.3)	8 (9.5)	12 (5.9)	1 (2.4)	45 (24.2)	10 (16.1)	81 (9.6)
Other‡	68 (28.2)	5 (21.7)	19 (22.6)	57 (28.1)	15 (35.7)	30 (16.1)	11 (17.7)	205 (24.4)
Marital status, n (%)								
Single	36 (14.9)	11 (47.8)	35 (41.7)	75 (36.9)	9 (21.4)	134 (72.0)	45 (72.6)	345 (41.0)
Married	138 (57.3)	5 (21.7)	25 (29.8)	60 (29.6)	21 (50.0)	10 (5.4)	1 (1.6)	260 (30.9)
Cohabiting	45 (18.7)	2 (8.7)	15 (17.9)	47 (23.2)	7 (16.7)	28 (15.1)	10 (16.1)	154 (18.3)
Divorced/separated/widowed	22 (9.1)	5 (21.7)	9 (10.7)	21 (10.3)	5 (11.9)	14 (7.5)	6 (9.7)	82 (9.8)
Sexual relationship status, n (%)								
Current partner	210 (87.1)	15 (65.2)	59 (70.2)	165 (81.3)	33 (78.6)	117 (62.9)	35 (56.5)	634 (75.4)
No current partner	31 (12.9)	8 (34.8)	25 (29.8)	38 (18.7)	9 (21.4)	69 (37.1)	27 (43.5)	207 (24.6)

*Borderline nuclear abnormalities and/or mild dyskaryosis.

†Data missing or <5 years of education.

‡Includes unemployed, self-employed, housewife/husband, long-term sick leave, and other.

CIN, cervical intraepithelial neoplasia; GW, genital warts; SD, standard deviation; VIN, vulval intraepithelial neoplasia.

### Psychosocial burden assessment (HIP questionnaire)

**Figure 2:**
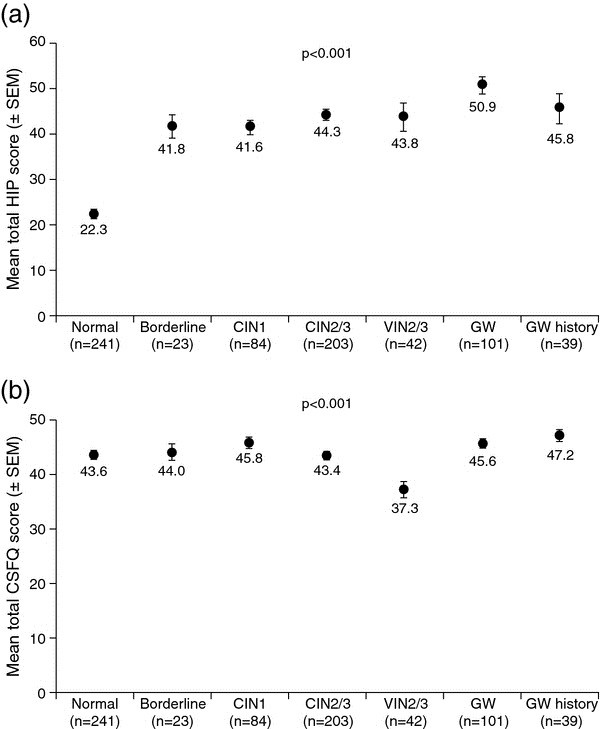
**Analysis of psychosocial burden and sexual function assessments (crude data, women only). (a)** Comparison of mean total HIP scores. Higher scores indicate greater psychosocial impact. **(b)** Comparison of mean total CSFQ scores. Higher scores indicate better sexual functioning. p value is from analysis of variance. Borderline, borderline nuclear abnormalities and/or mild dyskaryosis; CIN, cervical intraepithelial neoplasia; CSFQ, Change in Sexual Functioning Questionnaire; GW, genital warts; HIP, Human Papillomavirus Impact Profile; Normal, normal cervical cytology; SEM, standard error of the mean; VIN, vulval intraepithelial neoplasia.

Women with HPV-related disease had significantly higher mean total HIP scores than women with normal cervical cytology (Figure 2a, p < 0.001). Similar results were observed for all HIP dimensions (p ≤ 0.001, for all dimensions).

Women with GW had the highest mean total HIP score (50.9 [standard deviation, SD: 18.3] vs 22.3 [SD: 11.5] for women with normal cervical cytology), and had a significantly higher mean HIP score compared with women with VIN2/3 (50.9 [SD: 18.3] vs 43.8 [20.2], p = 0.044). Mean total HIP score was similar in women with GW and history of GW (50.9 [SD: 18.3] vs 45.8 [SD: 19.0], p = 0.166).

Mean dimension scores were similar for women with HPV-related disease except for women with GW, who had significantly higher mean scores compared with women with VIN2/3 and women with a history of GW for the following dimensions: partner issues and transmission (58.7 [SD: 27.9] vs 27.6 [SD: 25.4], p < 0.001 for VIN2/3 comparison; 58.7 [SD: 27.9] vs 35.0 [SD: 25.8], p < 0.001, for history of GW comparison); sexual impact (63.2 [SD: 32.5] vs 46.9 [SD: 30.9], p = 0.012, for history of GW comparison) and self-image (62.7 [SD: 25.3] vs 48.8 [SD: 22.9], p = 0.003, for VIN2/3 comparison).

### Sexual functioning assessment (CSFQ)

Women with HPV-related disease had similar mean total CSFQ scores compared with women with normal cervical cytology, except women with VIN2/3 who had a significantly lower mean total CSFQ score (Figure 2b, p < 0.001). Mean dimension scores for women with HPV-related diseases were similar to those for women with normal cervical cytology, except for women with VIN2/3 who reported worse scores for pleasure, desire/frequency, desire/interest and arousal/excitement.

Participants with GW or a history of GW reported similar sexual functioning: mean total CSFQ score for women: 45.6 (SD: 8.3) vs 47.2 (SD: 7.3), p = 0.323; mean total CSFQ score for men: 50.3 (SD: 5.9 vs 49.6 (SD: 4.8), p = 0.637, for GW and history of GW, respectively.

### QoL assessments

#### EQ-5D

##### Analysis for women with VIN2/3

The mean crude EQ-5D index score was 0.74 (SD: 0.27) and mean crude VAS score was 66 (SD: 19.9) for women with VIN2/3. They reported problems with anxiety/depression (64%), pain/discomfort (45%), usual activities (29%), mobility (21%) and self-care (7%).

**Figure 3:**
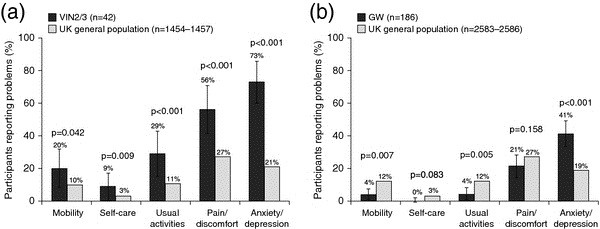
**Comparison of EQ-5D health domains in participants with VIN2/3 and GW versus UK population norms (weighted data). (a)** Percentage of women with VIN2/3 reporting some or extreme problems with EQ-5D dimensions compared with a sex-matched sample from the UK general population. p values are from *χ*^2^ tests and compare the VIN2/3 group with the UK general population. **(b)** Percentage of participants (men and women) with GW reporting some or extreme problems with EQ-5D dimensions compared with the UK general population. p values are from *χ*^2^ tests and compare the GW group with the UK general population. Error bars represent 95% confidence intervals. EQ-5D, European Quality of Life Index Version 5D; GW, genital warts; VIN, vulval intraepithelial neoplasia.

After weighting the data for the age distribution of the UK population, women with VIN2/3 had a significantly lower mean EQ-5D index score (0.72 [SD: 0.27] vs 0.89, p < 0.001) and mean VAS score (62 [SD: 18.9] vs 85, p < 0.001) than women in the UK general population. A significantly greater proportion of women with VIN2/3 reported problems with all dimensions of the EQ-5D index (mobility, self-care, usual activities, pain/discomfort and anxiety/depression) compared with women in the UK general population, particularly for the anxiety/depression dimension (73% [95% confidence interval [CI]: 59.6–86.4] vs 21%, p < 0.001) (Figure 3a).

##### Analysis for participants with GW

For women and men with GW, the mean crude EQ-5D index score was 0.84 (SD: 0.16) and 0.89 (SD: 0.17), respectively, and the mean crude VAS score was 75 (SD: 19.3) and 79 (SD: 15.5), respectively. Problems reported by women were with anxiety/depression (41%), pain/discomfort (27%), mobility (7%), usual activities (4%) and self-care (2%). Men reported problems with anxiety/depression (25%), pain/discomfort (22%), usual activities (6%) and mobility (2%).

After weighting the data for the age and sex distribution of the UK population, participants with GW had a similar mean EQ-5D index score compared with the UK general population (0.90 [SD: 0.13] vs 0.89, p = 0.633), but a significantly lower mean VAS score (78 [SD: 14.8] vs 85, p < 0.001). Overall, a significantly higher proportion of participants with GW reported problems with anxiety/depression compared with the UK general population (41% [95% CI: 32.7–49.3] vs 19%, p < 0.001). By contrast, significantly lower proportions of participants with GW reported problems with mobility (4% vs 12%, p = 0.007) and usual activities (4% vs 12%, p = 0.005) compared with the UK general population (Figure 3b).

When age-stratified analyses were performed on crude data, the youngest participants with GW had significantly lower mean EQ-5D index scores compared with UK norms (0.86 vs 0.94, p < 0.001 for 18–24-year-olds and 0.87 vs 0.93, p = 0.030 for 25–34-year-olds). Evaluation of mean VAS scores by age showed a statistically significant difference versus UK norms in the younger age groups only (76 vs 86, p < 0.001 for 18–24-year-olds; 80 vs 87, p = 0.004 for 25–34-year-olds; and 75 vs 87, p = 0.050 for 35–44-year-olds).

#### CECA

**Figure 4:**
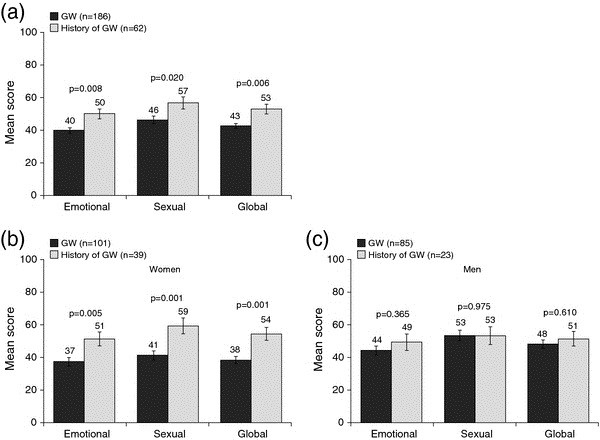
**Analysis of CECA scores (crude data). (a)** Comparison of mean CECA scores for male and female participants with GW versus those with a history of GW. **(b)** Comparison of mean CECA scores for women and **(c)** for men with GW versus those with a history of GW. Higher scores indicate a better health-related quality of life. p values are from the Student’s *t*-test and compare the GW group with the history of GW group. Error bars represent standard error of the mean. CECA, Cuestionario Especifico en Condilomas Acuminados; GW, genital warts.

Overall, participants with current GW had significantly lower mean CECA emotional, sexual and global scores than participants with a history of GW (non-current) (p = 0.008, p = 0.020 and p = 0.006, respectively; Figure 4a). After stratification by sex, similar differences were observed in women (Figure 4b) but not in men (Figure 4c).

## Discussion

At the time that the PasQual study was implemented, no published study had simultaneously used several patient-reported outcome instruments in individuals with HPV-related diseases to capture the impact of these diseases from a patient’s perspective. In the PasQual study, a significant negative psychosocial impact was found in women with a range of HPV-related diseases when compared with women with normal cervical cytology. Additionally, HPV-related external genital lesions (VIN2/3 and GW) were found to significantly impair HRQoL.

Differences in the age structure of the study groups reflected the epidemiology of the respective disease. The mean age of women with VIN2/3 was 45 years (range, 21–62 years). This is consistent with the fact that, historically, VIN2/3 and vulval cancer are associated with older age and long-term persistent infection with high-risk HPV, although both are now increasing in incidence in younger women [6]. By contrast, participants with GW or a history of GW had a mean age of 28 years (ranges, 18–60 and 19–52 years, respectively), reflecting the higher prevalence of GW in young adults than in older individuals [39].

The mean total HIP score for women with normal cervical cytology was 22.3, which was similar to mean total scores reported in the studies of Pirotta et al. (25.8) [40] and Wang et al. (28.2) [41], and about 8 points higher than that observed in the initial validation study for this instrument (14.4) [32]. The apparently elevated HIP scores for women with normal cervical cytology may indicate a negative impact of the cervical screening procedure itself, as observed in studies showing that women undergoing routine gynaecological examinations may experience pain or discomfort, embarrassment, fear, worry, nervousness and inconvenience [42,43]. However, in the absence of a defined scale linking scores to clinically relevant levels of impact, it is difficult to interpret absolute score values. In our study, women with normal cervical cytology were asked to complete the questionnaire when their test result was received. At this point, they were more likely to feel relieved and less likely to feel anxious than before receiving the result. Consequently, we consider that these women were a suitable reference group for the HIP questionnaire and CSFQ. In the absence of another adequate comparison group, men with a history of GW (non-current) were used as the reference group for men with current GW in analyses of the CSFQ.

Compared with women with normal cervical cytology, sexual functioning was significantly impaired only in women with VIN2/3, as assessed by the CSFQ. This finding may be partly related to age, as women with VIN2/3 tended to be older than participants in the other groups (mean age 44.8 vs 28.0–40.4 years, respectively). However, age is unlikely to be the only factor affecting sexual functioning. Women with VIN2/3 also had a highly impaired health state, as assessed by the EQ-5D index and VAS scores, compared with women in the UK general population, with particular detriments being observed in the anxiety/depression and pain/discomfort dimensions.

Women with current GW experienced the greatest negative psychosocial impact, as measured by the HIP questionnaire, while the impact was generally similar among women with other HPV-related diseases. These observations are consistent with reports of other studies that utilised the HIP questionnaire [32,40,41]. The mean total HIP score for women with current GW was 2.3-fold higher than the score for women with normal cervical cytology, which is consistent with the 1.8–3.7-fold higher scores observed in women with GW versus normal cervical cytology in previous studies [32,40,41]. Dimensions that were particularly affected in women with GW were sexual impact, self-image, and partner issues and transmission. These dimensions have also been previously identified as being of particular concern in women with GW [40,44]. Wang et al. observed similar results for sexual impact and self-image, but there was also a substantial impact of GW on worries and concerns and less impact on partner issues and transmission [41]. It is particularly notable that the impact of GW on psychosocial burden appears to be greater than that of diseases such as VIN2/3, which are considered to be more severe from a clinical perspective and have been shown to have a significant impact on sexual functioning in previous studies [30,45]. The high psychosocial impact of GW may be because they are visible and distressing and associated with discomfort and feelings of anxiety, depression, anger, fear of contagiousness, shame and embarrassment [22,23,25,46]. In addition, treatment of GW is long, painful and often unsatisfactory, with high recurrence rates [3].

As assessed by the EQ-5D, participants with GW reported problems with anxiety/depression more frequently than the UK general population. This is consistent with current knowledge regarding the experience of individuals with GW in whom much of the associated morbidity is psychological in nature [25]. By contrast, a lower proportion of participants with GW in our study reported problems with mobility and usual activities than the UK general population. This latter observation is likely to be due to the age difference between the two populations as participants with GW were younger overall than the UK general population. The observed impact on the anxiety/depression dimension (41% of participants with GW reported problems) is similar to that reported in the recent study of Woodhall et al. [24], in which 37% of participants with GW reported problems in this dimension, and consistent with the findings of other studies [20,22,23]. However, contrary to our observations, three of these studies reported an increased level of pain/discomfort [22-24] and one study reported a detrimental impact on usual activities [22] among individuals with GW when compared with general population samples.

Overall, participants with GW had a slightly impaired health state, as assessed by the EQ VAS only, compared with the UK general population. However, when focussing on young adults (18–34 years of age), in whom the prevalence of GW is the highest, both the mean EQ-5D index and VAS scores were significantly reduced compared with the UK population norms. In our study, the weighted mean EQ-5D index and VAS scores for participants with GW were 0.90 and 78, respectively, which are similar to those reported for the recent study of Woodhall et al. (0.87 and 77, respectively) [24]. A significant reduction in health state based on EQ-5D index and VAS scores in individuals with GW when compared with general population values has been reported in other studies [22,23], in which the average reductions in EQ-5D index score were 3.9 and 9.9 percentage points, respectively, and the average reductions in VAS scores were 13.9 and 6.0 percentage points, respectively.

Participants with current GW had a significantly impaired HRQoL, as assessed by CECA scores, compared with participants with a history of GW (non-current). A similar but lesser impact of GW on HRQoL has been demonstrated in men in a study using the initial 22-item CECA questionnaire [47]. When stratified by sex in the present study, the difference between GW and history of GW continued to be observed in women, but not in men. In the PasQual study, female participants with GW had significantly lower scores for the sexual domain of the CECA than those with a history of GW, whereas sexual functioning as assessed by the CSFQ was similar between participants with GW and a history of GW. This may seem contradictory, but it is important to note that whilst the CECA evaluates the psychological aspects of sexual life specifically related to GW, the CSFQ is a generic instrument that evaluates functional aspects of sexual life, which may explain these differences.

Despite several measures taken to try and increase study recruitment (e.g. extension of study period and reminders sent to participants), sample sizes were lower than planned in some groups in our study. For participants with borderline nuclear abnormalities and/or mild dyskaryosis, CIN1 and a history of GW, actual sample sizes were 23, 84 and 62 respectively, compared with the 200 planned for each group. Several reasons may explain the low numbers, including that many women screened for participation in the CIN1 group did not have histological confirmation of CIN1 and were therefore not included, in the other two groups, few individuals complied with the retrospective recruitment process and, in addition, individuals with a history of GW recruited prospectively often had other STIs, which was an exclusion criterion.

As each HPV-related disease is associated with specific demographic characteristics, the UK general population may not have been the best comparator to evaluate the representativeness of our study population with regard to each specific disease. Differences in the mean age between different HPV disease groups may have partially impacted comparison across groups, particularly for sexual functioning. The older age of the VIN2/3 group compared with other groups may have impacted the comparison of sexual functioning assessed by the CSFQ. Furthermore, the younger age of the GW group compared with the reference group could have masked a potential negative impact of GW on sexual functioning. For the CECA, participants with a history of GW were used as the reference group. Although this is not without limitations, we considered it to be the most appropriate comparator group as the CECA cannot be administered to individuals who have never experienced GW. The validation study for the CECA compared individuals with an initial diagnosis of GW with a group of individuals with persistent GW who were not receiving current treatment [34]. Furthermore, factors such as the number and size of lesions, which were measured in the validation study [34], were not considered in the current study. Finally, the study did not aim to collect clinical data. Thus, while we observed statistical differences in psychosocial burden and HRQoL between HPV disease groups, we could not assess the extent to which these differences were clinically meaningful.

## Conclusions

This study demonstrates that HPV-related non-cancerous and precancerous genital disease have a significant negative impact on psychosocial wellbeing and HRQoL. The health state of younger adults with GW, in whom the prevalence of GW is known to be greatest, was significantly impaired compared with UK normal values. VIN2/3 was found to have a negative impact on sexual functioning and women with VIN2/3 had a highly impaired health state compared with women in the UK general population.

## Abbreviations

CECA: Cuestionario Especifico en Condilomas Acuminados; CI: Confidence interval; CIN: Cervical intraepithelial neoplasia; CSFQ: Change in sexual functioning questionnaire; EQ-5D: European quality of life index version 5D; GW: Genital warts; HIP: HPV impact profile; HRQoL: health-related quality of life; HPV: Human papillomavirus; PasQual: Papillomavirus ASsociated QUAlity of Life; SD: Standard deviation; STI: Sexually transmitted infection; VAS: Visual analogue scale; VIN: Vulval intraepithelial neoplasia.

## Competing interests

SA-T and GD-F are employed by Sanofi Pasteur MSD. CC was employed by Sanofi Pasteur MSD until November 2009. HG is an employee of Mapi, a consultancy company commissioned by Sanofi Pasteur MSD to assist with the statistical analysis and interpretation of results of the PasQual study. AF received an honorarium for attending advisory board meetings related to this study and received financial support from Sanofi Pasteur MSD for attendance at a conference where she presented the study results. She has received honoraria from Sanofi Pasteur MSD and GlaxoSmithKline for attending advisory board meetings, an honorarium from GlaxoSmithKline for participating in conference activities, and financial support from Sanofi Pasteur MSD, GlaxoSmithKline and Roche to attend HPV-related conferences. AT has received financial support from Sanofi Pasteur MSD for attendance at a conference where she presented the study results. She has received honoraria from Sanofi Pasteur MSD and GlaxoSmithKline for attending advisory board meetings, payment for lectures from GlaxoSmithKline and Sanofi Pasteur MSD (the Wales College of Medicine also received funding from these lectures), and financial support from GlaxoSmithKline, Sanofi Pasteur MSD and Roche to attend meetings. The Wales College of Medicine received an honorarium for AT to attend advisory board meetings related to this study.

## Authors’ contributions

GD-F contributed to data analysis, participated in the interpretation of the study results, wrote the manuscript outline, and critically reviewed the manuscript drafts. CC was primarily involved in the design of the study, wrote the protocol, participated in the planning of statistical analyses, contributed to data analysis for the cross-sectional component of the study, and critically reviewed the manuscript drafts. HG designed and performed the statistical analyses, participated in the interpretation of the study results, and critically reviewed the manuscript drafts. SA-T coordinated the study activities, validated the results and study report, and critically reviewed the manuscript drafts. AT was involved in developing the concept and design of the study, patient enrolment (patient data acquisition), obtaining regulatory approval, overseeing recruitment, and critically reviewing the manuscript drafts. AF was the Chief Investigator for the study and was involved in developing the concept and design of the study, had responsibility for obtaining regulatory approval and overseeing recruitment, contributed to data analysis and interpretation of the study results, and critically reviewed the manuscript drafts. All authors approved the final version of the manuscript.

## Pre-publication history

The pre-publication history for this paper can be accessed here:

http://www.biomedcentral.com/1471-2458/13/1065/prepub
